# Evidence for an elementary process in bone plasticity with an activation enthalpy of 1 eV

**DOI:** 10.1098/rsif.2006.0172

**Published:** 2006-11-07

**Authors:** Himadri S Gupta, Peter Fratzl, Michael Kerschnitzki, Gunthard Benecke, Wolfgang Wagermaier, Helmut O.K Kirchner

**Affiliations:** 1 Department of Biomaterials, Max Planck Institute of Colloids and InterfacesMPI-KG Golm, 14424 Potsdam, Germany; 2 Univ Paris-SudUMR8182, Orsay, F-91405, France; 3 CNRSOrsay, F-91405, France

**Keywords:** bone plasticity, micromechanics of bone, deformation mechanisms, thermal activation, calcium mediated bonds

## Abstract

The molecular mechanisms for plastic deformation of bone tissue are not well understood. We analysed temperature and strain-rate dependence of the tensile deformation behaviour in fibrolamellar bone, using a technique originally developed for studying plastic deformation in metals. We show that, beyond the elastic regime, bone is highly strain-rate sensitive, with an activation volume of *ca* 0.6 nm^3^. We find an activation energy of 1.1 eV associated with the basic step involved in the plastic deformation of bone at the molecular level. This is much higher than the energy of hydrogen bonds, but it is lower than the energy required for breaking covalent bonds inside the collagen fibrils. Based on the magnitude of these quantities, we speculate that disruption of electrostatic bonds between polyelectrolyte molecules in the extrafibrillar matrix of bone, perhaps mediated by polyvalent ions such as calcium, may be the rate-limiting elementary step in bone plasticity.

## 1. Introduction

Bone is fracture resistant and shows large plastic deformation (Rho *et al*. 1998; Fratzl *et al*. 2004). Little quantitative information is available on the nature of the basic molecular level rearrangements under stress, which make this irreversible plastic deformation possible. Plasticity has to do with breaking and rearrangement of bonds, and bone is not an exception. Such processes can be helped by thermal activation (Kocks *et al*. 1975). The question is which bonds are breaking and how. In the case of metals, for example, the elementary process is known to be associated with the movement of lattice dislocations (Kocks *et al*. 1975), a process not likely to occur in the protein–mineral composite bone. As plasticity is a major factor reducing bone fragility, its origin is of the highest interest, both for the fundamental understanding of biological composites as well as to assess the possible origin of age-related fracture (Zioupos 2001) occurring without apparent change in the overall mechanical properties (Zioupos & Currey 1998). Owing to the hierarchical structure of bone (Rho *et al*. 1998; Weiner & Wagner 1998), length-scales ranging from the tissue level at 1–100 μm (Nalla *et al*. 2003) to the molecular scale (Mercer *et al*. 2006) have been considered to be responsible for the inelastic behaviour of bone. Indeed, the majority of studies do not use plasticity concepts but rather damage models (Carter & Caler 1985; Schaffler *et al*. 1994; Zioupos & Currey 1994; Reilly & Currey 2000) to understand the post-yield behaviour in bone, where damage means phenomena, such as microcracking and microfractures (Zioupos & Currey 1994; Zioupos 1999), observed with confocal scanning and light microscopy techniques.

In general, plastic deformation corresponds to the opening and reforming of bonds, leading to a permanent deformation. Thermodynamically, this corresponds to a movement over local energy barriers at the molecular level—leading to creep on a macroscopic scale—and can be described as an Arrhenius-type rate process (Schoeck 1965; Gibbs 1967). For permanent plastic deformation at a stress *σ* and temperature *T*, the macroscopic strain rate d*ϵ*/d*t* and the flow stress are related to each other through the microscopic activation energy barrier *H* and the volume *v* associated with the jump over the barrier, as

(1.1) $$\left(\frac{\text{d}\epsilon }{\text{d}t}\right)={\left(\frac{\text{d}\epsilon }{\text{d}t}\right)}_{0}\text{exp}\left(-\frac{H-v\sigma }{k_{\text{B}}T}\right).$$

The magnitude of *H* and *v*, thus obtained from macroscopic mechanical tests, give insight into the nature of the deformation mechanism at the molecular level. In metals and metal alloys, the activation volume for dislocation movement *v* can be written in terms of the Burgers vector for the basic dislocation step (Kocks *et al*. 1975), and thus provides information on the nature of the dislocation mechanism (Caillard & Martin 2003). This motivates us to see whether we could, similarly, quantify experimentally the length-scale and energy barrier associated with the elementary step at the molecular level for plastic deformation in bone.

## 2. Material and methods

Fibrolamellar bone from the periosteum of bovine femora (Gupta *et al*. 2005) was stretched to failure in a specially built tensile rig which enabled temperature control, from 4 to 50°C, in saline testing to keep the bone wet, and strain rates of up to 20–50% s^−1^ measured with video extensometry (figure 1 and electronic supplementary material). The tensile specimens had a gauge length of 6 mm and a cross-sectional area of 0.08 mm^2^ on average. Strain was measured from the percentage increase in separation of two markers on the bone imaged with a video camera. Temperature was typically kept constant during the test, although for a few measurements the temperature was changed abruptly in the yield region (see electronic supplementary material).

## 3. Results

*Activation volume*: to measure the activation volume *v* of plastic deformation, bone samples were stretched at constant motor velocity into the plastic yield region as shown in figure 2. When the sample was clearly in the zone of plastic deformation, the strain rate was reduced either once or several times (figure 2, inset). This change led to a reduction of the flow stress but, interestingly, the slope of the post-yield curve (linear hardening d*σ*/d*ϵ*) remained constant. From equation (1.1), the activation volume can be estimated as

(1.2) $$v=k_{\text{B}}T{\frac{\text{dln}(\text{d}\epsilon /\text{d}t)}{\text{d}\sigma }|}_{\text{T}}.$$

Using this differential method to estimate the activation volume leads to an average value of *v*=1.00±0.19 nm^3^ (*n*=8, error bars: standard deviations), implying that whatever (as yet unspecified) deformation processes occur in bone plasticity, the fundamental step is confined within a nanoscale volume. The activation volume is statistically independent (*p*>0.05) of stress, for a range of samples studied.

*Activation enthalpy*: to determine the height of the thermal activation barrier in bone deformation, numerous stretch-to-failure tests were done on bone tissue at strain rates varying from 1.4×10^−4^ to 2×10^−1^ s^−1^ and temperatures from 4 to 37°C (*n*=74 total; see table 1 in the electronic supplementary material for breakdown by strain rate and temperature). The lowest strain rates used were comparable to those previously used to measure fibrillar deformation using synchrotron radiation (Gupta *et al*. 2005), the intermediate strain rate was comparable to the rates obtained from physiological *in vivo* measurements (Robertson & Smith 1978; Burr *et al*. 1996) and close to strain rates of 0.6 s^−1^ typical for hip fractures (Courtney *et al*. 1996). The highest strain rates are a little below those rates at which the onset of brittle behaviour was observed (Mcelhaney 1966). For each sample, the yield stress *σ*_P_ was calculated as in figure 1*a*. Rewriting equation (1.1) as

(1.3) $$\sigma_{\text{P}}=\frac{1}{\nu }\left(H-X+Y\;\text{ln}{\left(\frac{\text{d}\epsilon }{\text{d}t}\right)}_{0}\right),\;\text{with}X=k_{\text{B}}T\;\text{ln}\left(\frac{\text{d}\epsilon }{\text{d}t}\right)\;\text{and}\;Y=k_{\text{B}}T,$$

we carried out a multiple linear regression of *σ*_P_ in terms of *X* and *Y*. An extremely significant (*p*<0.0001) correlation was found between the dependent (stress *σ*_P_) and independent variables (*X* and *Y*). The resulting fit parameters are: *H*=1.11±0.34 eV; *v*=0.64±0.07 nm^3^; and (d*ϵ*/d*t*)_0_=1.11×10^9^ s^−1^ (3.00×10^6^–4.09×10^11^ s^−1^), and the plane fit is shown in figure 3*a*. Representing our data in terms of *σ*–*T* graphs, as usual in analyses of thermally activated plasticity (Kocks *et al*. 1975), we show mean value and standard deviation of the yield stress for several values of temperature and strain rate in figure 3*b*. The yield stress decreases with increasing temperature for all the three strain rates. The broken lines show how equation (1.3) predicts that the yield stress *σ*_P_ would vary as a function of temperature at a given strain rate (using the fitted parameters from figure 3*a*).

The discrepancy between the activation volumes obtained from the global survey of yield stress data (0.64±0.07 nm^3^) and from the differential data of various flow stresses (1.00±0.19 nm^3^) is not surprising. The former refer to specimens with unmodified microstructure, at the onset of plastic deformation, the latter to microstructures already modified by plastic deformation. Such subtleties amount to strain and stress dependence of the activation volume (Kocks *et al*. 1975) and are beyond the concern of present bone research. We therefore take the latter value as confirmation of the former.

## 4. Discussion

To summarize, we find that plastic deformation in bone is characterized by a very small activation volume *ν* of the order of 1 nm^3^ and an activation enthalpy of the order of *H*=1.1 eV. This activation enthalpy is smaller than typical covalent bond energies (C–C bond approx. 3.6 eV) but much larger than hydrogen bonds (approx. 40 meV). The Gibbs free energy *G*(*σ*)=*H*−*vσ* is the free energy to be supplied during one activation event in the plastic deformation of bone. The applied stress *σ* increases the probability of the irreversible deformation occurring, by doing work against a certain basic volume of deformation *v*. *H* and *v* carry information on the energy barrier needed to go from the undeformed to the deformed state, but they give no information about the kinetics of this process.

The small activation volume suggests that the elementary process corresponds to the breaking of just a few spatially confined bonds. In metal plasticity, which is controlled by the dynamics of dislocations (Schoeck 1965; Gibbs 1967; Kocks *et al*. 1975), the activation volume is the area of slip × the Burgers vector (Kirchner 2006). In the case of bone, the small size of the activation volume is likely owing to a confinement of the soft organic matrix (which is likely to flow) between nanometre-sized particles. Size effects on mechanical properties are well known from the science of materials strength as seen, for example, in recent work (Uchic *et al*. 2004; Espinosa *et al*. 2005). Our recent *in situ* diffraction results (Gupta *et al*. 2005, 2006) suggest that after the onset of macroscopic plasticity, only elastic deformation is retained within fibrils and plastic deformation occurs between them.

The magnitude of the activation enthalpy *H*≅1.1 eV suggests that the bonds being broken are not likely covalent. Hydrogen bonds, having energy of 40 meV each, would have to break in large numbers (approx. 50) simultaneously to provide the necessary energy, but such a situation is inconsistent with the small activation volume of up to 1 nm^3^. As a consequence, the most likely types of bonds are charge interactions between molecules in the extrafibrillar space. It is not known which these molecules are, but substantial amounts of non-collageneous molecules, such as proteoglycans (Scott 1992), osteopontin (Sodek *et al*. 2000) or fetuin A (Heiss *et al*. 2003), are present in the bone matrix. These (mostly negatively) charged molecules (or any combination of them) could be responsible for forming a plastic ‘glue’ between fibrils. The existence of such a ‘glue’ has recently been proposed following force spectroscopy experiments (Thompson *et al*. 2001) and it was shown that the occurrence of bond breaking and reforming was related to the presence of calcium ions (Fantner *et al*. 2005, 2006). The energy associated with these ‘sacrificial bonds’ is consistent with the activation enthalpy of 1 eV found here (Fantner *et al*. 2006). Recently, it has also been shown that the deformation of polyelectrolyte capsules is associated with breaking a group of neighbouring charge interactions on polyelectrolyte segment (Leporatti *et al*. 2001). The activation enthalpy was shown to be *ca* 1 eV in this case and the activation volume (corresponding to the size of the polyelectrolyte segment) was also in the range of *ca* 1 nm^3^ (Leporatti *et al*. 2001). Breaking and reformation of bonds has also been found in the hemicellulose matrix between cellulose fibrils in the cell wall during plastic deformation of wood (Keckes *et al*. 2003).

Thermodynamics of plastic deformation interprets the pre-exponential factor (d*ϵ*/d*t*)_0_ as a product of an attempt frequency *ν*_0_ and the deformation *ϵ*_0_ caused by each activated event. The latter quantity *ϵ*_0_ depends on the process controlling strain (dislocation density, obstacle density, etc.). It is difficult to put a precise value for the attack frequency *ν*_0_, but it must be of the order of vibrations present in the medium. The phonon spectrum of bone has never been measured, but presumably, it must be similar to the spectrum of type I collagen found in tendon (Middendorf *et al*. 1995). The latter shows several broad maxima between 1×10^13^ and 6×10^13^ s^−1^, which have been attributed to various localized modes. Given the fact that possible localized modes at the interface between fibrils must be low-frequency ones otherwise the interfacial entropy would be negative, it is not unreasonable to assume a value of *ν*_0_=10^12–13^ s^−1^. Our fit results provide a value lower than this range (1.11×10^9^ s^−1^) but, unsurprisingly in light of the discussion above, this is the fit parameter that showed a substantial error (approx. 30% in the logarithm) in the multiple linear regression, leading to a possible range of values from 3.00×10^6^ to 4.09×10^11^ s^−1^.

Damage has been associated with the post-yield behaviour, but the nature of the damage is unclear (Carter & Caler 1985; Schaffler *et al*. 1994; Zioupos & Currey 1994; Zioupos *et al*. 1994; Zioupos 1999; Reilly & Currey 2000). The damage is believed to be related to the formation of microcracks or smaller defects at weak interfaces such as between old and new bone packets in trabecular bone, between lamellae in lamellar cortical bone or between osteons and interstitial bone (Lakes & Saha 1979; Braidotti *et al*. 2000; Diab *et al*. 2006; Peterlik *et al*. 2006). In the present work, we try to avoid the effect of these weak interfaces, both by preparing samples whose cross-section is of the order of the width of single fibrolamellar bone packets (see electronic supplementary material) and by studying this relatively parallel fibred tissue in tension. Nevertheless, the breaking of bonds (within an activation volume of *ca* 1 nm^3^) can be regarded as damage at the supramolecular level which has, however, the capability of self-healing by the reformation of the bonds. An indication that this is the case is seen in cyclic tensile loading of our samples in the inelastic regime (figure S6 in electronic supplementary material). The initial slopes of the loading segments are similar, indicating recovery at the material level. However, the progressively lower yield stress for successive cycles implies that the recovery may be incomplete, due to damage at the nanoscale level. As such, the post-yield behaviour reported here has more resemblance to plastic deformation in metals than to damage as observed in many composite materials.

The results of this paper are in excellent agreement with the previous work showing that the deformation in bone might be associated with (calcium-dependent) sacrificial bonds (Thompson *et al*. 2001; Fantner *et al*. 2005, 2006) and with independent work demonstrating that the plastic deformation occurs in a thin ‘glue’ layer between fibrils (Gupta *et al*. 2005, 2006). The picture which emerges is that plastic deformation is controlled by an elementary process where segments of molecules in the interfibrillar layer are connected by charge interactions with a total energy of 1 eV in a volume of 1 nm^3^ (corresponding to the volume of the rigid molecular segments which move in a coordinated fashion). These results are summarized in the model drawn in figure 4.

In conclusion, our mechanical tests on bone established a high sensitivity of the macroscopic plastic deformation to the strain rate and temperature. By putting our results in the scheme of thermally activated processes controlling bone plasticity, quantitative results can be obtained on the length-scale and energy associated with bone plasticity mechanisms at the molecular level. The fundamental processes involved in plastic deformation are localized to within 1 nm^3^, and with energy of the order of 1 eV. We speculate that these processes are localized in a small fraction of the bone tissue—the extrafibrillar matrix—and correspond to the disruption of calcium-mediated ionic bonds between the long and irregular chains of molecules constituting this matrix.

## Figures and Tables

**Figure 1:**
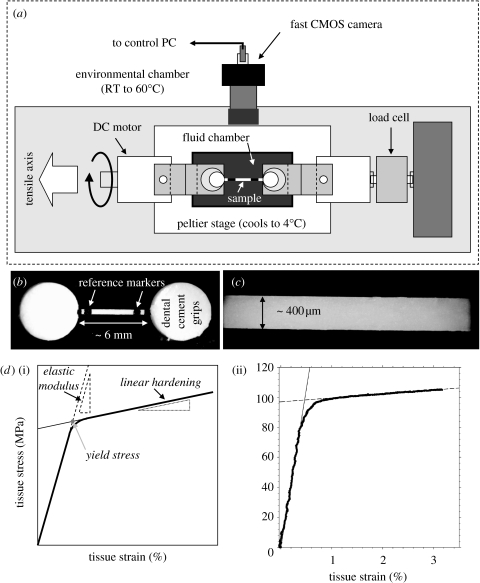
(*a*) Overview of the tensile test set-up. (*b*) Light microscope image of a test sample between two cylindrical dental glue grips. (*c*) Larger magnification view of the sample, showing the homogeneous structure. (*d*(i)) a schematic of a typical stress–strain curve, with the definitions of elastic modulus, linear hardening and yield stress indicated; (ii) a representative stress–strain curve taken with strain rate 1.5×10^−4^ s^−1^ at 16°C, showing the elastic modulus, linear hardening and yield stress.

**Figure 2:**
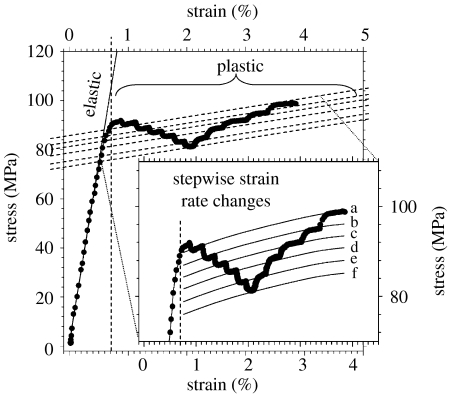
Measurement of the activation volume *v* in bone plasticity: the plot shows a uniaxial tensile test at constant temperature *T*=296 K with six stepwise reductions in motor velocity from 10 to 0.1 μm s^−1^ (*a*→*f*≡10 μm s^−1^→5 μm s^−1^→2 μm s^−1^→1 μm s^−1^→0.5 μm s^−1^→0.2 μm s^−1^→0.1 μm s^−1^) and subsequent increases. The differential changes in stress with differential changes in strain rate can be used to compute the activation volume (equation (1.2)).

**Figure 3:**
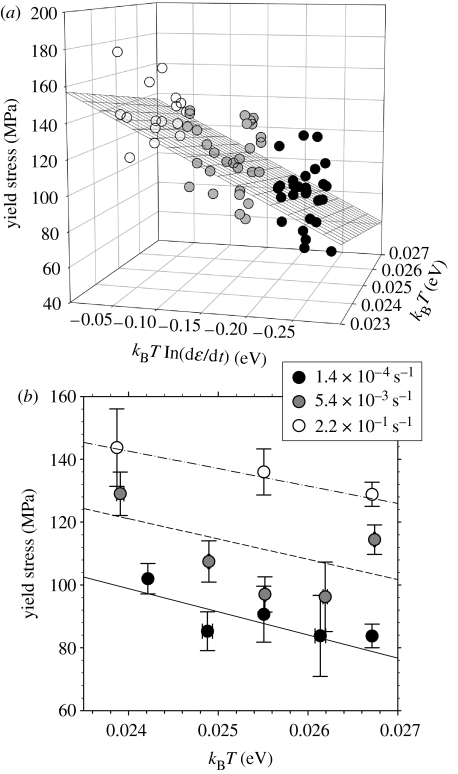
Measurement of the activation enthalpy *H* of bone plasticity: a set of samples (*n*=74) are stretched to failure in tension, at three strain rates and at least three temperatures at each strain rate, from 277 to 310 K, and the yield stress *σ*_P_ measured. Plot (*a*) shows the yield stress *σ*_P_ as a function of *k*_B_*T* and *k*_B_*T* ln(d*ϵ*/d*t*). Black grid lines show the results of the linear plane fit $\sigma_{\text{P}}(T,\text{d}\epsilon /\text{d}t)=(H/v)+(1/v)k_{\text{B}}T\;\text{ln}(\text{d}\epsilon /\text{d}t)-(\text{ln}{\left(\text{d}\epsilon /\text{d}t\right)}_{0}/v)k_{\text{B}}T$. Plot (*b*) shows the same three-dimensional plot in the *σ*_P_–*k*_B_*T* plane, giving the average stress (error bars: standard deviations) for a given strain rate and temperature. Lines are predictions based on model fit in (*a*), at the given three strain rates: dash-dotted lines and white circles≡2.2×10^−1^ s^−1^; short dashed lines and grey symbols≡5.4×10^−3^ s^−1^; solid lines and black symbols≡1.4×10^−4^ s^−1^. Note that the fact that we view (*a*) at an angle almost 90° to the effective viewing direction in (*b*) is deliberate, chosen to give the reader the perspective from two orthogonal directions.

**Figure 4:**
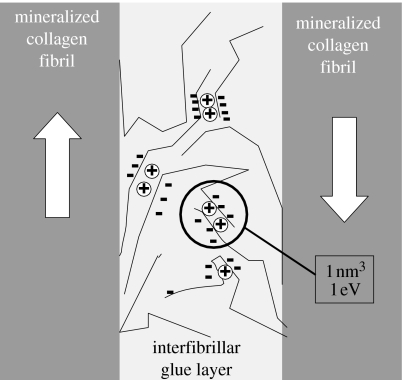
Bond breaking within the thin extrafibrillar matrix (glue layer) between mineralized fibrils controls bone plasticity, based on results in this work as well as previous papers proposing deformation by sacrificial bonds (Thompson *et al*. 2001; Fantner *et al*. 2005; Hansma *et al*. 2005) and demonstrating a shear deformation of the glue layer (Gupta *et al*. 2005). Long chains of molecules (possibly negatively charged polyelectrolytes like osteopontin (Sodek *et al*. 2000), fetuin A (Heiss *et al*. 2003) or proteoglycans (Scott 1992), or combinations of those) are interacting by charges, probably with the help of cations such as calcium (circles). Charges located on a given segment will have to be broken together giving rise to the observed activation enthalpy of *ca* 1 eV within a typical volume of 1 nm^3^. The arrows indicate the movement of the collagen fibrils giving rise to shear in the matrix layer (Gupta *et al*. 2005). Mineral particles are not explicitly drawn, but present in the fibrils as well as in the interfibrillar space.

## Supplementary Material

Tensile Testing of Bone in a Temperature Controlled Fluid Cell with High Speed Video Extensometry Here we give an overview of the experimental setup for tensile testing of wet fibrolamellar bone in a temperature range 4° to 50°C. The video extensometry procedure used to measure the strain in a non - contact method is described, including the special procedure used for high strain rates (<10 % s-1). In addition, representative cyclic tensile loading curves are shown, to demonstrate similar values of loading and reloading slopes
