# Supplementary material for: Evidence for an elementary process in bone plasticity with an activation enthalpy of 1 eV
Source: J R Soc Interface. 2006 Nov 7;4(13):277–82. doi: 10.1098/rsif.2006.0172 (PMC2220070; doi:10.1098/rsif.2006.0172)
Supplement: Supplementary file 1 [file rsif20060172s01.pdf]

Electronic Supplementary Material

Materials and Methods:

Sample preparation: Fibrolamellar bone was sectioned from the periosteum of a bovine femur (18 month old ox) as described previously (Gupta et al., 2005, Gupta et al., 2006), using a Buehler ISOMET low speed saw (Buehler Gmbh, Düsseldorf, Germany) and a Leica SP1600 inner hole saw (Leica Microsysteme Vertrieb Gmbh, Bensheim, Germany). Samples had dimensions of ~ 11 mm × 0.4 mm × 0.2 mm, with the long (tensile) axis of the sample parallel to the bone long axis, which in turn is approximately parallel to the direction of the mineralized collagen fibrils (Figure S3 in (Gupta et al., 2005)). Samples were kept wet throughout the preparation process. After production, the samples were wrapped in cotton gauze soaked in phosphate buffered saline (PBS) solution (Sigma Aldrich Gmbh, Germany) and stored at -20 °C.

Tensile testing: Samples prepared as described above were thawed at room temperature. Two reference markers were placed on the sample with indelible ink for video extensometric measurements of tissue strain. Sample dimensions were measured with a pressure sensitive micrometer and by taking digital images under a stereomicroscope (Leica MZ7.5 stereo light microscope with add-on 2.0 × objective, Leica Microsysteme Vertrieb Gmbh, Bensheim, Germany). The ends of the samples were coated in

cyanoacrylate. Using specially designed Teflon moulds, the ends of the samples were embedded in water resistant dental ionomer cement (3M ESPE, Seefeld, Germany). The dental cement moulds act as grips to hold the sample in the tensile grips without the excessive force and stress concentrations created by mechanical clamps (Gupta et al., 2006, Gupta et al., 2005). After embedding the effective gauge length of the sample (distance between the dental cement grips) was reduced from 11 mm to about 6 mm.

A specially designed tensile tester was used to make the mechanical tests at a controlled temperature and strain rate. The design was shown in Figure 1 (main text), and a photograph of the tester is given in Figure S1. Grips with cylindrical hollows were used to hold the cylindrical cement ends. The grips themselves were attached by pivoting pins to the motor axis. This construction enabled self – alignment of the sample into the direction of the tensile axis, in case it was inserted slightly off – axis to start with, and was observed by us to be a major factor in obtaining long regions of plastic (yield) deformation. Tensile strain was applied by the movement of a DC – encoder motor (M-126.PD1, Physik Instrumente, Karlsruhe, Germany). This stage was used in preference to the M – 126.DG stage used in previous experiments (Gupta et al., 2006, Gupta et al., 2005) because it could move with a maximal velocity of 15 mm/s, compared to a maximal velocity of 5 mm/s for the M – 126.DG. The stage was controlled via a C – 862 Mercury™ II controller (Physik Instrumente, Karlsruhe, Germany), which is connected via a RS-232 cable to the controlling PC. Force was measured by a 12.5 kg load cell with 10 V DC excitation and temperature compensation, connected to an amplifier (A. L.

Design Inc., Buffalo, NY, USA). The signal from the load cell was read by a DAQ PCI card (6052-E, National Instruments, München, Germany) in the controlling PC.

To keep the sample wet during testing, a fluid bath/immersion chamber was filled with PBS (arrows in Figure 1, main text). The grips are designed to dip into the fluid bath during testing, keeping the sample fully immersed. A small glass slide was placed on the upper side of the immersion chamber, to eliminate optical distortion due to meniscus formation on the surface of the PBS solution. To control the temperature, the fluid chamber was fixed with a thin layer of thermoconductive paste onto a Peltier cooling element, which was controlled to a temperature controller and power supply. A thermocouple was inserted into the fluid chamber near the position of the sample to read the sample temperature, and the output was read by the DAQ PCI card. The entire tensile stage was mounted inside an environmental chamber (Kleinbrutschrank Heraeus® B 15, Thermo Electron Corporation, Karlsruhe, Germany), which could provide an ambient temperature ranging from room temperature to 60 °C. To obtain a given temperature above room temperature (typically ~ 23 °C) during the tensile test, we adjusted the analog control of the environmental chamber to approximately the value desired (with an accuracy of 1 °C) and then used the Peltier cooling element with attached electronics to fine – tune the temperature at the fluid chamber / sample to the precise value. We were usually able to achieve temperature control to within 0.2 °C of the desired temperature.

To measure the tissue strain, we used video extensometry to measure the distance between two reference markers on the sample. A CMOS camera (BCi4-LS-M-20-P with PCI – LS interface, Vector International, Leuven, Belgium) with a PCI – LS interface (Vector International, Leuven, Belgium) with a high frame acquisition rate was used. Usually, anisotropic regions of interest (ROIs) were acquired of the anisotropic sample itself (Figure S1), in order to economize the amount of data transferred. The reference markers provided a strong contrast (black) against the white bone material. To find the positions of the markers, line profiles of pixel gray levels along the direction of tensile load were read. Absolute values of the derivatives of the gray level line profiles were calculated, and the mean position of the peaks in the derivative spectra of the pixel gray levels (corresponding to the transition between black and white areas on the image) were used as the positions of the edges of the markers.

To measure the distance between the markers, we took special care to correct for any artifacts arising from a slight non-parallelism of the sample edges to the edges of the orthogonal ROI. Specifically, before the test was started, the user provides (via mouse input) an initial guess for the edge of the sample. The self written edge detection routines locate this edge more precisely, and then the distance between the two reference markers is measured at a user given offset from the edge. At each step in the data acquisition process (i.e. at each step when the stress, video image, and temperature is measured) the sample image is analyzed to find the optimal line describing the edge of the sample with respect to the orthogonal ROI, and the new distance between the reference markers is

measured at the same predefined offset from this edge. This procedure corrects for any artifacts arising if the sample should rotate by a couple of degrees during the testing, perhaps during the initial prestressing or alignment phase.

The software interface was made using self written software in Labview version 7.0 (National Instruments, München, Germany), and a special DLL library was written for communication to the CMOS camera in fast transfer mode (pseudo DMA, where DMA means Direct Memory Access). We had two different modes for testing, which we denote “online” and “offline” analysis. In the first “online” analysis mode, images taken by the camera of the sample are analyzed along with the load cell and temperature sensor signal, and the stress, strain, and temperature displayed in real time during the test. For the fast strain rates (> 10 %/s) the rate of data transfer meant that a DMA mode transfer was the only possible method. In such a mode, the motor is prescribed a given distance to move at a (high) velocity. The test then runs, and in a short interval of time (1 – 10 s) the sample is broken. During the test, a continuous stream of analog data (temperature and load) is read from the DAQ card. The synchronization of the video images and the analog data stream (load cell and temperature sensor) was achieved by a common triggering signal sent from a commercial function generator (FG-7202, Voltcraft®, Conrad Electronic GmbH, Germany). Subsequent to the test, the camera releases from its buffer a series of images acquired during the short test. After the test (in “offline” mode) the user scans through these to find the images corresponding to the interval where the sample was

stretched and broken. The images in this subselection are then analyzed to calculate tissue strain.

Cyclic Loading of Bone

Cyclic loading of our test samples in tension into the post yield region support the idea that the damage at the supramolecular level is partially recoverable. As shown in Figure S6, the initial slopes on the loading curves of bone is only slightly smaller than the slope in the elastic region, and approaches a constant value after the first unloading cycle. However, the yield stress is lower for subsequent cycles, as indicated by the red dashed line going from the upper left to lower right.

Supplementary Table:

n	v[mm/s]	T[°C]	strain rate[%/s]	d(strain rate)	Stress[MPa]	d(stress)
7	1.00E-03	8.0	1.33E-02	1.01E-03	102.0	4.8
6	1.00E-03	15.7	1.04E-02	1.32E-03	85.3	6.2
6	1.00E-03	23.0	1.68E-02	1.10E-03	90.7	8.9
5	1.00E-03	30.3	1.27E-02	1.68E-03	83.8	12.9
5	1.00E-03	37.0	2.26E-02	2.15E-03	83.8	3.8
6	2.00E-01	4.4	6.97E-01	8.23E-02	129.0	6.9
4	2.00E-01	15.9	8.14E-01	7.36E-02	107.5	6.6
6	2.00E-01	23.1	4.63E-01	5.30E-02	97.0	5.6
6	2.00E-01	30.9	5.15E-01	6.09E-02	96.2	11.1
7	2.00E-01	37.3	6.05E-01	5.13E-02	114.4	4.7
4	1.50E+01	4.0	1.85E+01	2.32E+00	143.7	12.3
6	1.50E+01	23.0	2.37E+01	2.33E+00	136.0	7.3
6	1.50E+01	37.0	2.47E+01	1.85E+00	128.9	3.8

Table S1: Summary of mechanical data at different temperatures and motor velocities. Values shown are from tensile tests on n = 74 bovine fibrolamellar bone samples at 3 constant applied nominal motor velocities and different temperatures. At a given temperature T and motor velocity v, n denotes the number of samples tested, and d(strain rate) and d(stress) refer to standard error of mean of strain rate and yield stress respectively. The data is the same as shown graphically in Figure 3(b) in the main text.

Supplementary Figure Legends:

Figure S1: Image of the micro tensile tester (a), with the sample mounted inside the self aligning grips (b). Cold light source illuminates the sample, which is inside a phosphate buffered saline bath, which in turn sits on a Peltier cooling element, and the whole setup is enclosed inside a temperature chamber (not shown; surrounds the tester shown in (a)).

Figure S2: (a) The left schematic shows an idealized stress – strain curve, where following an initial linear elastic region, the bone enters the region of irreversible plastic deformation. For simplicity, the linear hardening (small positive slope of the stress – strain curve in the post yield region) is ignored. If a sudden change (increase) in strain rate is applied to the sample, then the flow stress increases. The magnitude of the increase in stress can be used to measure the activation volume of bone (Equation (2), main text). (b) The right schematic shows an idealized stress – strain curve, where the strain rate is kept constant throughout but the temperature is discontinuously changed (decreased) at a point in the region of plastic deformation. The decrease in temperature leads to an increase in plastic flow stress given on the plot in terms of the microscopic parameters.

Figure S3: A sudden change (decrease) in strain rate during plastic deformation of bone at constant temperature leads to a sudden change (decrease) in the plastic flow stress of bone: Experimental validation of the scheme in Figure S2 (a). A sample at room temperature (23 °C) is stretched at a motor velocity of 0.01 $mm.s^{-1}$ into the yield region, at which point the velocity is reduced to 0.0005 $mm.s^{-1}$. Concurrent with the reduction of strain rate, the yield stress drops by about 16 MPa. When the velocity is changed back to 0.01 $mm.s^{-1}$, the yield stress returns to the higher level. Dashed lines are guides to the eye meant to represent the stress – strain curves at the two strain rates.

Figure S4: A sudden change (decrease) in temperature leads to a sudden alteration (increase) in stress during plastic deformation of bone: Experimental validation of the scheme in Figure S2 (b). A sample initially at ~ 37 °C, is stretched into the region of plastic deformation (solid line shows the slope of the stress – strain curve in the elastic regime, giving the elastic modulus). At tissue strain ~ 1.0 %, a change (reduction) in temperature to ~ 20 °C is made by adding cooler PBS into the fluid cell (vertical lines in (a) and (b)), but via an electronic feedback system, the strain rate is maintained constant at a value of $2.5 \times 10^{-5}\ s^{-1}$. The low strain rate used here enabled highest control in maintaining a constant strain rate from the video extensometry signal during the change in temperature. The fact that the temperature change was almost exactly at 1.0 %, and that the vertical line intersects the elastic modulus line fit also at 1.0 % in (a) (top of plot) is a coincidence, and was not so designed in the experiment.

Figure S5: Differential strain rate measurements of the activation volume *v* (Equation (2)) plotted as a function of stress just before reduction of strain rate, for a range of strain

rate changes. Data from $n = 8$ samples are shown, with mean value of 1.00 ± 0.19 nm^3 (error bars: standard deviation). Dotted line shows the statistically nonsignificant ($p > 0.05$) decrease of activation volume with stress.

Figure S6: Multiple cyclic loading of bone in tension. Dark blue lines: loading, light gray: unloading curves. Blue solid lines are linear regression fits to the initial slope of each loading segment. As a visual aid, the reduction in yield stress between loading cycles is indicated by the red dashed line from upper left to lower right.

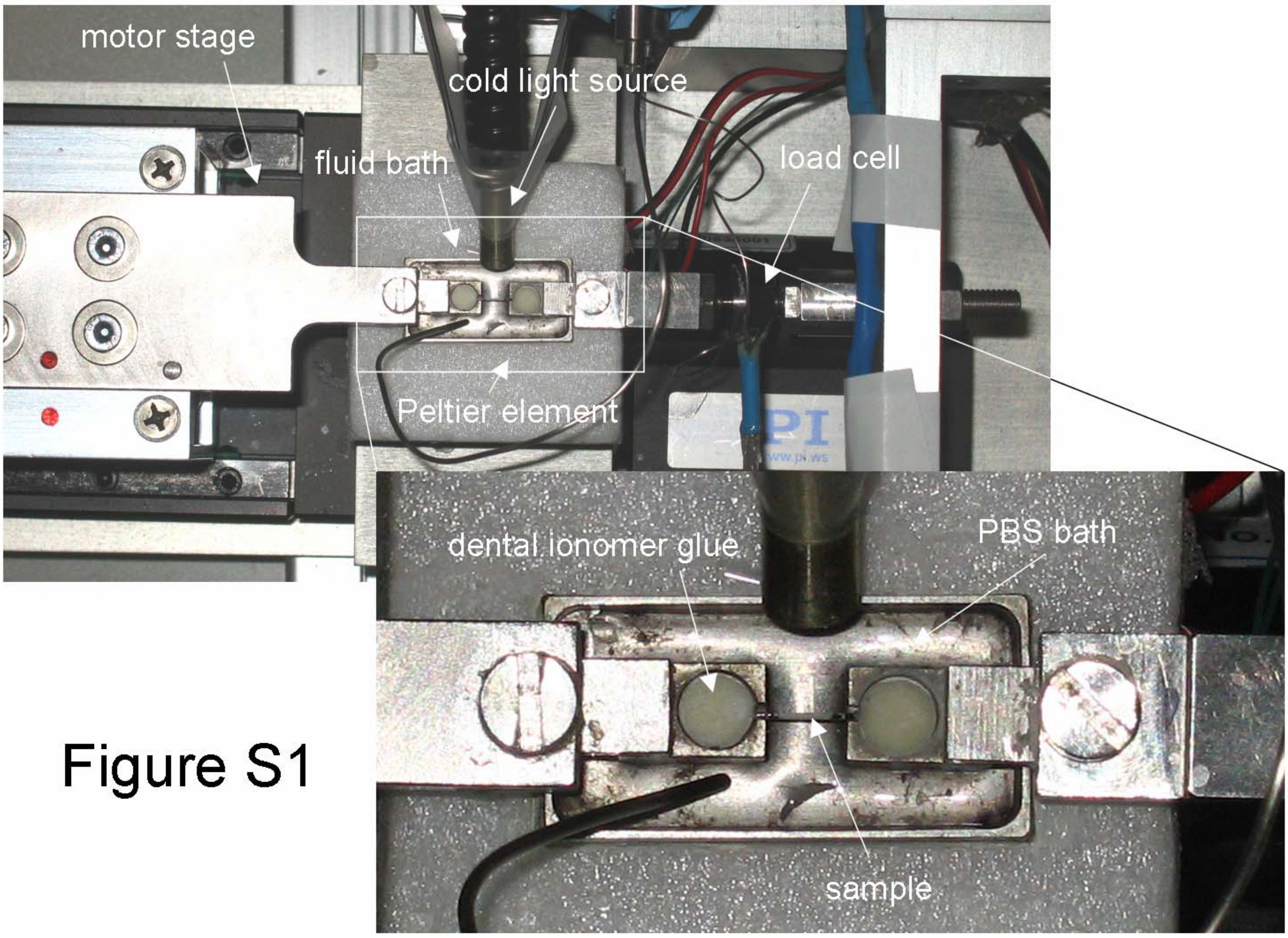
motor stage
cold light source
fluid bath
load cell
Peltier element
dental ionomer glue
PBS bath
sample

Figure S1

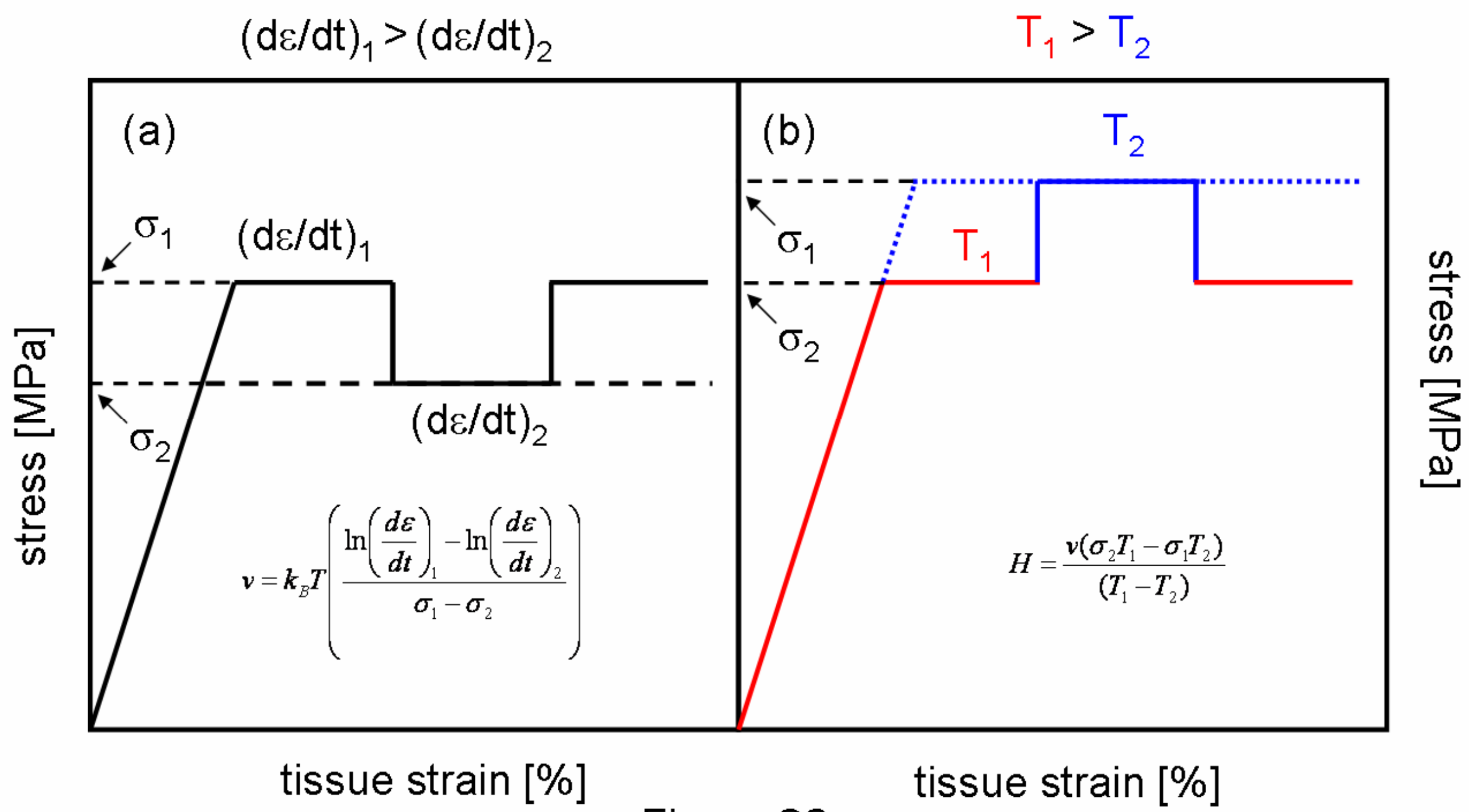

(dε/dt)1 > (dε/dt)2
T1 > T2
(a)
(b)
σ1
σ2
(dε/dt)1
(dε/dt)2
T1
T2
stress [MPa]
tissue strain [%]
$v = k_B T \left(\frac{\ln\left(\frac{d\varepsilon}{dt}\right)_1 - \ln\left(\frac{d\varepsilon}{dt}\right)_2}{\sigma_1 - \sigma_2} \right)$
$H = \frac{v(\sigma_2 T_1 - \sigma_1 T_2)}{(T_1 - T_2)}$

Figure S2

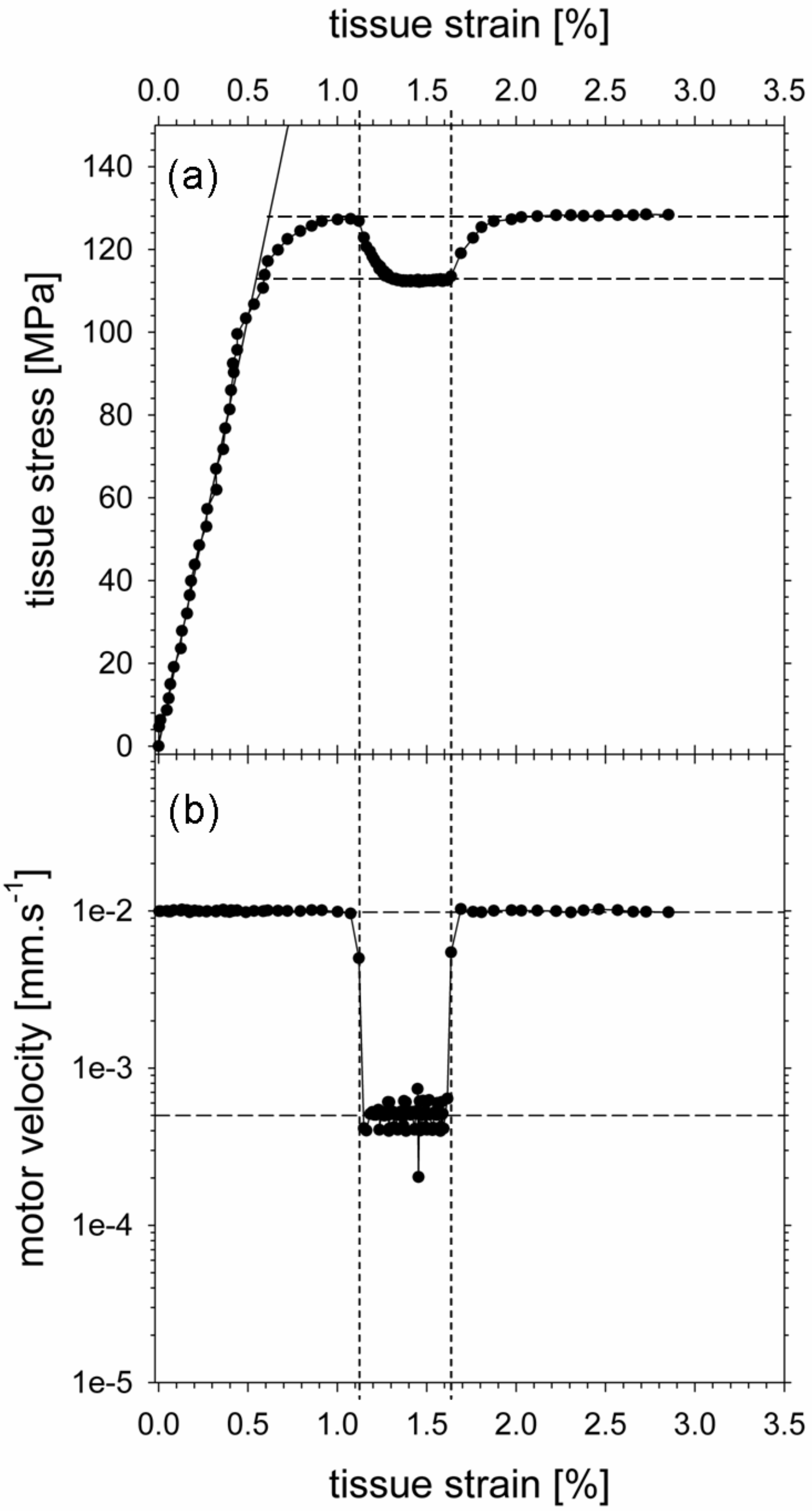

tissue strain [%]
0.0
0.5
1.0
1.5
2.0
2.5
3.0
3.5
(a)
tissue stress [MPa]
0
20
40
60
80
100
120
140
(b)
motor velocity [mm.s-1]
1e-2
1e-3
1e-4
1e-5
tissue strain [%]

Figure S3

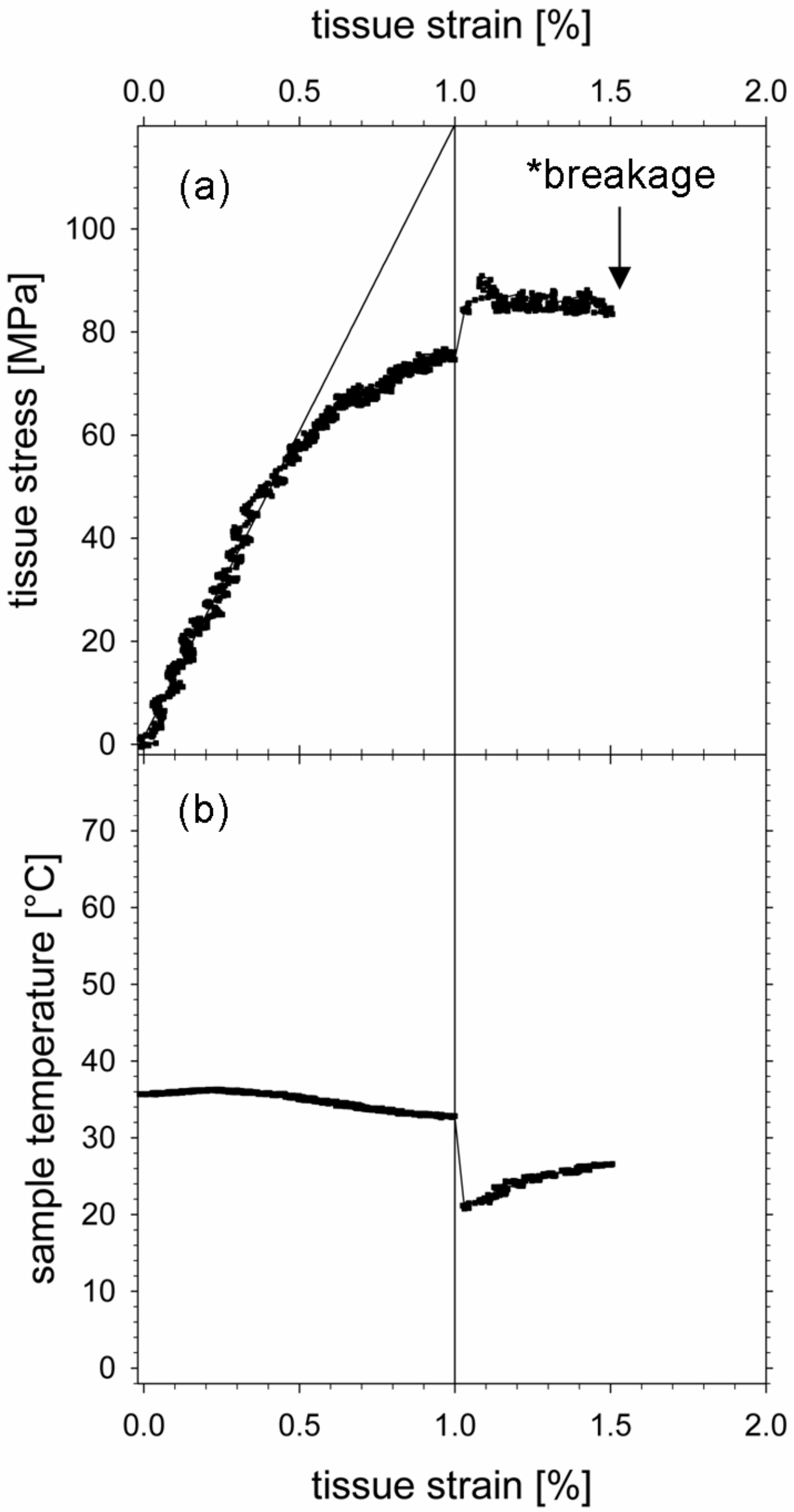

tissue strain [%]
0.0
0.5
1.0
1.5
2.0
(a)
*breakage
100
80
60
40
20
0
tissue stress [MPa]
(b)
70
60
50
40
30
20
10
0
sample temperature [°C]
0.0
0.5
1.0
1.5
2.0
tissue strain [%]

Figure S4

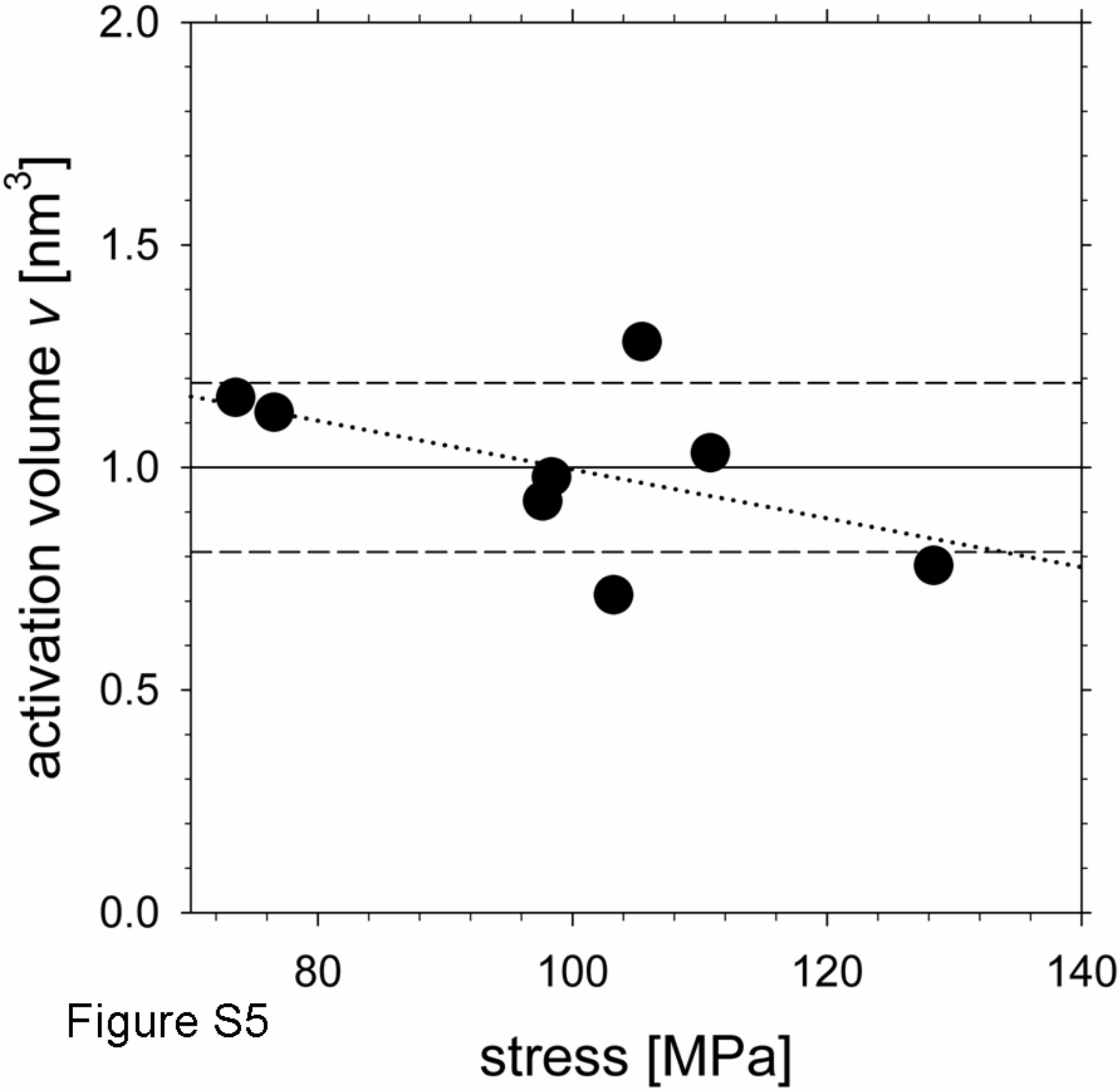
2.0
1.5
1.0
0.5
0.0
activation volume v [nm3]
80
100
120
140
stress [MPa]

Figure S5

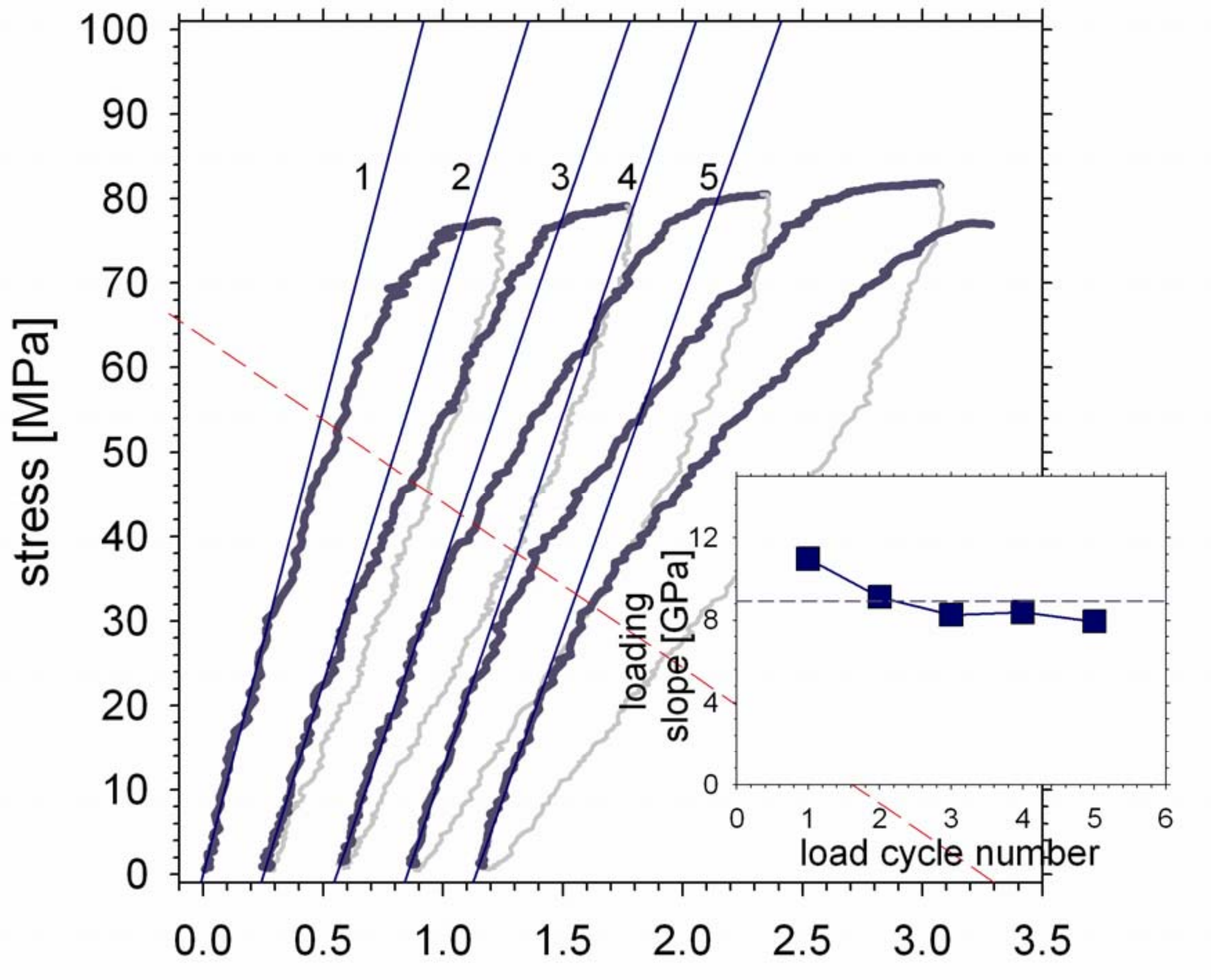

stress [MPa]
100
90
80
70
60
50
40
30
20
10
0
0.0
0.5
1.0
1.5
2.0
2.5
3.0
3.5
1
2
3
4
5
loading
slope [GPa]
12
8
4
0
0
1
2
3
4
5
6
load cycle number
strain [%]

Figure S6
